# A New Paradigm in Cardiac Regeneration: The Mesenchymal Stem Cell Secretome

**DOI:** 10.1155/2015/765846

**Published:** 2015-05-05

**Authors:** Clara Gallina, Valentina Turinetto, Claudia Giachino

**Affiliations:** Department of Clinical and Biological Sciences, University of Turin, Regione Gonzole 10, Orbassano, 10043 Turin, Italy

## Abstract

The potentialities to apply mesenchymal stem cells (MSCs) in regenerative medicine have been extensively studied over the last decades. In the cardiovascular disease (CVD) field, MSCs-based therapy is the subject of great expectations. Its therapeutic potential has been already shown in several preclinical models and both the safety and efficacy of MSCs-based therapy are being evaluated in humans. It is now clear that the predominant mechanism by which MSCs participate in heart tissue repair is through a paracrine activity. Via the production of a multitude of trophic factors endowed with different properties, MSCs can reduce tissue injury, protect tissue from further adverse effects, and enhance tissue repair. The present review discusses the current understanding of the MSCs secretome as a therapy for treatment of CVD. We provide insights into the possible employment of the MSCs secretome and their released extracellular vesicles as novel approaches for cardiac regeneration that would have certain advantages over injection of living cells.

## 1. Introduction

Cardiovascular diseases (CVD), which are the leading cause of morbidity and mortality worldwide, account for approximately 30% of all deaths, with nearly half resulting from myocardial infarction (MI) [1]. Unlike some other organs, the heart has a limited ability to regenerate, and dysfunction resulting from significant cardiomyocyte loss under pathophysiological conditions, such as MI, can lead to heart failure (HF). Unfortunately, even with the advance in pharmacological therapies and improvements in mechanical support devices, for patients with end-stage HF, heart transplantation remains the main alternative, but the limited availability of donor organs renders it insufficient.

Adult mesenchymal stem or stromal cells (MSCs) are nonhematopoietic cells capable of self-renewal and multilineage differentiation into various tissues of mesodermal origin. They could reside in virtually all postnatal organs and vascularized tissues [2], but they are mainly found in bone marrow (BM-MSCs), where they constitute 0.001%–0.01% of the bone marrow cells. Upon isolation, MSCs are a heterogeneous cell population characterized by their capacity to adhere to plastic, develop as fibroblast colony-forming-units, and differentiate into three cell lineages: osteocytes, chondrocytes, and adipocytes. After* in vitro* culture expansion, they are positive for the cell surface markers CD73, CD90, and CD105 and negative for CD11b, CD14, CD34, CD45, and human leukocyte antigen- (HLA-) DR [3]. Due to their multipotency and paracrine effect, MSCs are ideal candidates for regenerative medicine and immunotherapy [4].

In the present review we will discuss the literature regarding application of MSCs for cardiac regeneration, presenting a concise summary about cell-based therapy followed by a deeper analysis of MSCs paracrine effects via secreted factors and extracellular vesicle release.

## 2. Mesenchymal Stem Cells Participate in Cardiac Repair via Different Mechanisms

Cell-based cardiac repair offers the promise of rebuilding the injured heart [5]. Mobilization of cells with endogenous cardiac regenerative potentials, as occurs after injury to other organs like skeletal muscle and liver, would represent the ideal strategy to restore cardiac function after MI. Indeed, there is emerging evidence for a certain level of cardiomyocyte turnover in the mammalian heart. Several types of cardiac resident stem cells (CSCs) and cardiac progenitor cells (CPCs) were isolated and identified in the adult heart, including c-Kit^+^ and Sca1^+^ cells [6]. In general, it has been proposed that CSCs can differentiate into the three major heart lineages: myocardial, smooth muscle, and epithelial cells [7]. The search for CPCs and CSCs that can readily differentiate within damaged tissue and differentiate into functioning cardiomyocytes continues with success [8].

Meanwhile, regenerative therapy using bone marrow-derived mononuclear cells (BM-MNCs) and MSCs has shown considerable promise. Between 2002 and 2005 the first stem-cell-based clinical trials for MI initiated using unfractionated and highly heterogeneous adult BM-MNCs. Despite initial positive results indicating safety of BM-MNCs transplantation and some beneficial effects on heart function, subsequent overall analysis of these first-generation trials revealed several inconsistencies possibly due to the differences in trial design, outcome evaluation, and cell isolation, thereby preventing general conclusions [9] and sending researchers back to the bench to elucidate strategies to overcome these limitations [10]. Recent clinical trials have utilized more homogenous BM-MSCs populations that were isolated and expanded in culture. Among them, the first clinical trial for acute MI using human adult MSCs (hMSCs) was a randomized, double-blind, placebo-controlled, dose-escalation study of intravenous cell injection that provided pivotal safety and provisional efficacy data [11].

In order to contribute to cardiovascular repair,* in vitro* expanded MSCs can act through different mechanisms. MSCs can transdifferentiate into cardiomyocytes, as seen in a study employing female pigs which underwent experimental acute MI and 3 days later received transendocardial injections of allogeneic male bone marrow-derived MSCs; cell engraftment and differentiation into cardiomyocytes and vascular structures were documented [12]. However, another work showed that there was no evident engraftment of hMSCs in murine infarcted hearts few weeks after MI [13]. Accordingly, only about 2% of the administered MSCs remained localized in the normal pig heart 2 weeks after coronary infusion and no evidence was obtained indicating MSC differentiation to cardiomyocytes [14]. In conclusion, MSCs transdifferentiation into contractile cardiomyocytes seems to be inefficient [15] and to occur only in the presence of native cardiomyocytes [16–18].

Another possible mechanism is fusion of MSCs with native cells, even though this phenomenon was very infrequently observed, ruling out any substantial involvement in MSCs-mediated cardiomyocyte regeneration [16, 19].

MSCs-induced stimulation of endogenous CSCs via direct cell-cell interaction [16] and MSCs-dependent paracrine signaling [17, 20] remain two other possible cardiac regeneration mechanisms. There is still significant debate about whether MSCs need to engraft at the target site of injury or can exert their effects systemically. Cell engraftment might increase the potential for cell-cell contact besides increasing the release of immunomodulatory and trophic factors* in situ*. However, the ischemic microenvironment is characterized by oxidative stress and inflammation, hostile conditions that pose a serious problem for MSCs survival. Experiments in small animals have shown that MSCs do not persist well inside the graft environment and if there is or no incorporation into the host tissue, most of the cells are lost within a month [18]. Failure of MSCs attachment in the ischemic microenvironment might be exacerbated by reactive oxygen species- (ROS-) dependent inhibition of cell adhesion to the extracellular matrix (ECM) components [21], an event that could hinder the physical interaction of MSCs with endogenous CSCs.

Thus, a more likely explanation for MSCs-mediated cardiovascular repair is via exocytosis of a complex secretome made up of growth factors, cytokines, and other signaling molecules in the form of both released factors and extracellular vesicles [22], which may generate a microenvironment suitable to support regenerative processes, induce angiogenesis, and protect against further tissue death [23].

**Figure 1:**
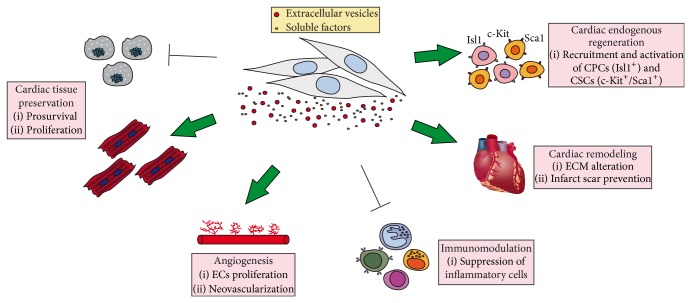
*The broad repertoire of MSCs secretome has considerable potential for the treatment of CVD*. In the context of myocardial protection, potential therapeutic mechanisms established by MSCs secretome include tissue preservation, angiogenesis, beneficial cardiac remodeling, anti-inflammatory responses, and finally promotion of endogenous regeneration of the heart. CPCs: cardiac progenitor cells; CSCs: cardiac stem cells; ECs: endothelial cells; ECM: extracellular matrix.

The proposal that the paracrine activity of MSCs would be central for their therapeutic efficacy is supported by recent preclinical studies demonstrating improved cardiac function upon infusion of cytokines or conditioned medium (CM) in the absence of cell transplantation.  Figure 1 summarizes some of the most characterized aspects of cardioprotection in which MSCs secretome has been involved, and they will be extended in the following section.

## 3. Secretome-Based Therapeutic Efficacy of Mesenchymal Stem Cells for Cardiovascular Disease

### 3.1. Released Factors: Cytokines and Growth Factors

#### 3.1.1. Cardiac Tissue Preservation and Remodelling

The first studies implying paracrine activity of MSCs as central to their cardiac therapeutic efficacy date back almost ten years ago and evidenced trophic, prosurvival, and antiapoptotic effects (Figure 1).

Among other studies, Takahashi and colleagues assessed that MSCs-derived cytokines were able to preserve myocardial contractile capacity, inhibit apoptosis of cardiomyocytes, and allow the formation of new vessels in damaged tissues [24]. Gnecchi et al. demonstrated that MSCs overexpressing Akt gene (Akt-MSCs) exposed to hypoxia produced a CM that was able to prevent death of isolated adult rat ventricular myocytes, as documented by reduced morphologic evidence of necrosis or apoptosis and attenuated release of Caspase 3. Moreover, the same CM significantly reduced infarct size in a rodent infarct model [25]. In the specific context of MI, Iso et al. [13] compared the gene expression profiles of cultured hMSCs with those of freshly isolated CD133^+^ bone marrow stem cells recently being evaluated as a candidate cell population for treating MI in patients [26]. To clarify the implications of the array data, authors ran ELISAs for several protective secreted factors on serum-free CM from the hMSCs donor used to treat the infarcted mice [13]. Results showed that cultured hMSCs expressed mRNAs for antiapoptotic and matrix-mediating factors, the majority of them expressed to a greater extent in hMSCs than in freshly isolated CD133^+^ cells [13]. In particular, expression of IL-6 and LIF family members was 40–200-fold higher. Several mRNAs for matrix-mediating factors, such as matrix metalloproteinase- (MMP-) 2 and inhibitors such as (TIMP)-1,2 and matricellular proteins (Thrombospondin-1 and Tenascin C), were also highly expressed in hMSCs [15]. Overall, the results of microarray analyses demonstrated that cultured hMSCs expressed mRNAs for a variety of secreted factors that may be cardioprotective and separative [13].

Follow-up functional genomics studies then revealed that Secreted Frizzled Related Protein 2 (Sfrp2), a member of the Wnt signaling pathway, was significantly upregulated in Akt-MSCs-CM compared to control MSCs and its attenuation by siRNA silencing abrogated Akt-MSCs-mediated cytoprotective effects [27]. Further studies indicated that a novel secreted protein, hypoxic induced Akt regulated stem cell factor (HASF), upregulated in Akt-MSCs subjected to normoxia or hypoxia, may mediate survival effects in isolated hypoxic cardiomyocytes via PKC-*ε* signaling by blocking activation of mitochondrial death channels [28].

In another study it was shown that ablation of TNF receptor 1 (TNFR1) but not TNFR2 in mouse MSCs enhanced protection following their infusion in the injured myocardium and correlated with reduced levels of ventricular TNF-*α*. Based on this evidence it was postulated that MSCs paracrine effects and associated cardioprotection are likely mediated by TNFR2 [29]. Furthermore, using a swine model of acute MI, Nguyen et al. have shown that intracoronary injection of concentrated MSCs-derived growth factors significantly reduced cardiac troponin-T elevation and improved echocardiographic parameters [30].

#### 3.1.2. Angiogenesis

Transplanted MSCs can release soluble factors contributing to neoangiogenesis inside the heart (Figure 1). Accordingly, Li et al. demonstrated that GATA-4 overexpression increased both MSCs survival and their angiogenic potential in the injured myocardium [55]. In particular, rat BM-MSCs (rMSCs) transduced with GATA-4 demonstrated increased secretion of proangiogenic factors and, when transplanted in infarcted rat hearts, these cells were able to increase blood vessel formation and decrease infarct size [55]. Furthermore, murine BM-MSCs infected with the proangiogenic miR-126 showed enhanced expression of the notch ligand delta-like (Dll)-4, with subsequent increased secretion of angiogenic factors and higher resistance to hypoxia. When these cells were injected in a murine MI model they displayed increased survival in the injured tissue, thereby improving cardiac function and microvessel density [56]. Finally, Timmers et al. collected hMSCs secretions using a clinically compliant protocol and intravenous treatment with this CM of a swine model of MI was found to increase capillary density and preserve cardiac function, probably by increasing myocardial perfusion [57]. When authors began to evaluate the gene expression profiles for proteins produced by cultured hMSCs and implicated as potential angiogenic/arteriogenic factors, secreted cysteine-rich protein 61 (Cyr61) was found to be a key molecule for the observed effects according to immunodepletion experiments [35]. This protein was also abundantly expressed in the cellular proteome of murine MSCs as shown by Liquid Chromatography Mass Spectrometry (LC-MS)/MS, western blot, and immunofluorescence [35]. These findings suggest that Cyr 61 has a key role and contribution in promoting angiogenesis, during regeneration and repair of injured tissues [35].

#### 3.1.3. Immunomodulation

The functional ability of MSCs to modulate the immune system seems to play a role in almost all the effects attributed to these cells via three major mechanisms: cell-to-cell contact, production of inhibitory molecules, and induction of regulatory T-cells [58]. MSCs have been shown to suppress inflammatory reactions in a variety of different disease states or damaged tissues [17, 59–61] and, interestingly, the proposed mechanisms seem to be specific for the cause of inflammation. In the case of a mouse model of induced asthma, for example, MSCs suppressed Th2-mediated inflammation in a manner that involved TGF-beta secretion and the activation of IL-4- and IL-13-induced STAT6 pathway [59], while in the environment set by interstitial lung disease, inflammation was suppressed by MSCs through mechanisms involving TNF-alpha and IL1R [60].

In a mouse model of acute MI inflammation was suppressed through MSCs-dependent production of the anti-inflammatory factor TNF-*α*-induced protein 6 (TNAIP6 or TSG-6); in particular, this molecule was associated with suppression of the excessive inflammatory response consequent to permanent ligation of the anterior descending coronary artery (LAD), decrease of the proteolytic damage to the heart and the subsequent fibrotic scarring, and increase in cardiac function [17].

#### 3.1.4. Cardiac Endogenous Regeneration

Another recognized effect of MSCs secretome is the promotion of a regenerative microenvironment inside the injured tissue (Figure 1), with direct evidence from studies on MSCs-CM [26, 35, 58]. To date, several disease models have been used, among them chronic kidney disease [61], lung [62], and liver injury [63], demonstrating that MSCs-CM alone is sufficient to mediate long lasting therapeutic effects.

In the CVD concern, there is strong evidence that MSCs secrete trophic factors that induce* in vitro* proliferation of endogenous CPCs. For example, Nakanishi et al. highlighted that rMSCs-CM promoted proliferation and migration of isolated CPCs and prevented their apoptosis when subjected to hypoxia and serum starvation. Furthermore, conditioned CPCs also showed upregulated expression of cardiomyocyte-related genes such as beta-myosin heavy chain (beta-MHC) and atrial natriuretic peptide (ANP) [64]. Another study using a hamster model of HF demonstrated a novel noninvasive therapeutic regimen via the direct delivery of MSCs into the skeletal muscle bed [40]. Intramuscularly injected MSCs or CM significantly improved ventricular function 1 month after their administration; myocyte regeneration was evidenced by an approximately twofold increase in the expression of cell cycle markers (Ki67 and phosphohistone H3) and an approximately 13% reduction in mean myocyte diameter. Finally, increased circulating levels of hepatocyte growth factor (HGF), leukemia inhibitory factor (LIF), and macrophage colony-stimulating factor (M-CSF) were associated with the mobilization of c-Kit^+^, CD31^+^, and CD133^+^ progenitor cells and a subsequent increase in myocardial c-Kit^+^ cells [40]. In addition, MSCs secrete mobilizing factors such as HGF, LIF, SDF-1, SCF, and VE-Cadherin and, thus, the transplanted MSCs secretome could also be beneficial for mobilization and homing of host MSCs [40].

### 3.2. Extracellular Vesicles

Extracellular vesicles (EVs) is a term recently proposed by György et al. [65] to describe membrane-limited cellular components, discernible by their size and composition and released by several cell types. Current research has focused principally on microvesicles (MVs) and exosomes, although other vesicular structures can be secreted, among them microparticles and apoptotic bodies [65]. MVs, initially described in blood [66], have a size between 100 nm and 1 *μ*m and derive from the detachment of cytoplasmic protrusions with a process that depends on the increase of intracellular calcium concentration and subsequent enzyme activation (e.g., calpain) and cytoskeleton reorganization. MVs expose high amounts of phosphatidylserine and specific protein markers, such as integrins, selectins, or CD40 ligand [67]. On the other hand, exosomes have a size ranging between 30 and 100 nm and originate upon fusion of multivesicular endosomes with the plasma membrane. These vesicles are released by exocytosis [68] by most cell types including immune cells [69, 70], cancer cells [71], and MSCs [72]. Their membranes may expose unique proteins that reflect their cellular source, besides being rich in tetraspanins (CD9, CD63, and CD81) and heat-shock proteins. Notably, exosomes can contain numerous proteins and lipids as well as messenger RNA (mRNA) and microRNAs (miRNAs) responsible for intercellular signaling.

A recent systematic review on animal studies of various kinds of injury highlighted that MSC-derived MVs are strongly associated with improved organ function [73]. Initial demonstrations came from models of renal disease, where MVs were shown to protect the kidney from toxic injury by producing factors that limited apoptosis and enhanced proliferation of endogenous tubular cells [74]. In the context of CVD, recent studies suggest that the therapeutic effect of MSCs-derived paracrine action is in large part due to secreted EVs [75] and exosomes seem to be principally involved in these effects, as reported by the majority of the literature data in this concern. In particular, in 2007 Timmers et al. highlighted that the CM of human embryonic stem cell-derived MSCs injected in a porcine model of myocardial I/R was able to limit infarct size and improve systolic function via probable reduction of TGF-*β* signaling and apoptosis [76]. Further fractionation analyses then revealed that marked cardioprotection was mediated by CM components with a size between 100 and 220 nm [76]. Subsequent works from the same group showed that highly purified exosomes isolated from CM of the same MSCs had a hydrodynamic radius of 55–65 nm and induced significant cardioprotection when injected in a murine MI model [77]. Notably, this effect was mediated by intact but not lysed exosomes, with specific increase of ATP levels, Akt and GSK-3*β* phosphorylation and reduction of oxidative stress, phosphorylation of c-Jun, and inflammatory response in the reperfused myocardium [51].

Besides promising results, however these works do not underline the precise effectors that mediate protection in the heart. In this concern, it has been shown that exosomes can also deliver nucleic acids, like microRNAs (miRNAs). A recent study showed that preconditioned murine BM-MSCs released exosomes enriched with miR-22. These vesicles were highly internalized by cocultured cardiomyocytes and prevented their apoptosis via interaction of miR-22 with methyl CpG binding protein 2 (Mecp2). Finally, delivery of these enriched exosomes into mice subjected to MI led to marked reduction of fibrosis [46]. Furthermore, exosomes derived by rMSCs transduced with GATA-4 contained high levels of several miRNAs, among them miR-221 and miR-19a; interestingly, these EVs were able to reduce apoptosis of ischemic cardiomyocytes via miR-221-dependent inhibition of p53-upregulated modulator of apoptosis (PUMA), a subclass of the Bcl-2 protein family [53], but also via miR-19a-associated inhibition of PTEN that resulted in activation of Akt and ERK pathways [45].

Altogether, these data support the assumption that MSC-dependent paracrine function inside the heart might be due not only to freely released soluble factors, but also to secreted exosomes that can deliver a large amount of peptides or other molecules protecting them from eventual degradation and facilitating their uptake inside cells. According to previous findings correlating exosome internalization with microenvironmental/intracellular acidity [78], it was proposed that a mechanism of MSC-derived exosome delivery might be favoured by low pH typical of ischemic cardiomyocytes [72]. Furthermore, several authors suggested that MSCs-exosomes therapy could be more attractive than direct cell transplantation to treat CVDs, due to avoided surgery-associated injury or risk of cell differentiation into other cell types, such as osteoblast or adipocytes or even tumor-like cells. However, further studies will be needed to elucidate the specific signaling molecules delivered by exosomes that subsequently elicit protective mechanisms in the injured myocardium [28, 72].

## 4. Secretome Proteomic Profiling

### 4.1. Released Factors

Due to these encouraging preclinical results, the MSCs-CM has become a subject of intensive proteomic profiling, in order to identify the released factors that might be applicable in regenerative medicine. Analysis of MSCs secretome was recently enabled mainly for the extensive development in protein separation techniques, mass spectrometry, immunological methods, and bioinformatics [79]. The* in vivo* secretome profiling, relying on the analysis of body fluids or the interstitial solution that directly surrounds the cells, would seem to most accurately reflect cell secretions in their native microenvironment. In such an* in vivo* approach, capillary microdialysis devices or ultrafiltration probes can be used to collect body fluids [80]. Unfortunately, MSCs represent only a small subpopulation among the various cell types within the tissue; thus the analysis of their secretome inside body fluids or tissue explants is extremely difficult. Hence, studies of MSCs secretions are currently performed under* in vitro* conditions via collection of media conditioned by cells mostly for 12–48 h of culture [79].

Since 2003, when the first proteomic analysis of human BM-MSCs secretory counterpart was undertaken [81], more than 30 additional studies have been published, showing the growing interest in the MSCs secretome. These studies have identified numerous candidate modulators for paracrine effects and cell-mediated/inflammatory suppression (Table 1). Sarojini et al. published a study in which secretome derived from mouse stem cell cultures stimulated chemotaxis of human fibroblasts [34]. 19 secreted proteins, including ECM structural proteins, collagen processing enzymes, pigment epithelium-derived factor (PEDF), and cystatin C, were identified. Interestingly, PEDF was recognized as one of the most abundant proteins in the CM; immunodepletion and reconstitution experiments further revealed that this protein was the predominant chemoattractant for fibroblasts [34]. Many studies identified secretion of proangiogenic factors, including Adrenomedullin [13], Cyr61 [35], and IL-1 [39], and LV remodelling attenuation was observed through secretion of factors promoting either vasculogenesis [31, 82] or endothelial tube formation [33]. Other MSCs secreted factors promoted mobilization of cardiac stem/progenitor cells [13, 38] or BM-derived progenitor cells [32, 40]; specifically, Secreted Frizzled Related Protein (SFRP 2) promoted MSC self-renewal and survival [41, 42]. Cardiomyocytes survival improvement was mediated by at least four different secreted factors [13, 36, 37]. Finally, anti-inflammatory factors like soluble TNFR1 [43] and TSG-6 [17] were identified (Table 1).

### 4.2. Extracellular Vesicles

Isolation of EVs relies on their purification from supernatants of cells grown in absence of serum [83] through ultracentrifugation [84], ultrafiltration [85], or immunoprecipitation technologies using antibody loaded magnetic cell beads [86]. To date, several thousand proteins and RNAs have been described in EVs purified from various cell types or biological fluids (see Table 1 for a selection of such molecules specifically engaged in cardiovascular repair). These studies allowed the identification of a common set of components, mainly associated with the biogenesis or structure of vesicles or proteins specific for the cell origin or physiopathological status (for review, see [87]).

EVs from MSCs specifically express CD13, CD29, CD44, CD73, and CD105 [50, 88, 89] and other surface molecules that are characteristic of the tissue origin [90]. MSCs-EVs also contain nucleic acids, both mRNA and noncoding RNA. The mRNAs present in EVs are representative of the multiple differentiation and functional properties of MSCs, including transcripts related to several different cell functions such as the control of transcription, cell proliferation, and immune regulation [74, 90]. Among the noncoding RNAs contained in released MSCs-EVs there are selected patterns of miRNAs [91, 92], small noncoding RNAs that regulate gene expression posttranscriptionally by targeting specific mRNAs. Interestingly, these miRNAs could be subsequently transferred to target cells [91, 93] and were functionally active, as evidenced from their ability to downregulate proteins targeted by selected transferred miRNAs [91, 93–95]. Gene ontology analysis of the molecules targeted by the highly expressed miRNAs in MSCs-derived EVs revealed genes involved in multiorgan development, cell survival, and differentiation [91].

More recently, Lai et al. [47] focused on the proteome of exosomes to identify candidate proteins or protein complexes that could drive their therapeutic efficacy in ameliorating myocardial I/R injury in a mouse model of MI. Clustering of these proteins according to their functions had suggested that MSCs exosomes have the potential to drive many biological processes, an observation consistent with the reported efficacy of MSCs in treating a myriad of diseases encompassing as cardiovascular (e.g., acute myocardial infarction, end-stage ischemic heart disease, or prevention of vascular restenosis) and noncardiovascular diseases [96]. The proteome of MSCs exosomes obtained by three independent experiments and analysed by mass spectrometry and cytokine array identified 857 proteins that were distributed over a wide array of biochemical and cellular processes such as communication, structure and mechanics, inflammation, exosome biogenesis, tissue repair, and metabolism [96].

In some studies the specific exosome-contained molecules able to mediate the protective effects on myocardium were identified: miR-19a promoted the cardioprotective Akt/ERK signaling [45], miR22 reduced apoptosis and improved ischemic injury [46], and 20S proteasome subunits exerted cardioprotection through degradation of misfolded proteins [47] (Table 1). In other cases, angiogenesis promotion [48–50], oxidative stress inhibition [51], and hypoxic signal pathway inhibition [52] were recognized as cardioprotective functions contained in MSCs exosomes, yet the responsible molecules have not been identified yet (Table 1).

Finally, MSCs microvesicles were found to improve cardiomyocytes survival through miR221 [53] and to increase endothelial cell proliferation and blood flow recovery [54] (Table 1).

## 5. Conclusions

Regenerative medicine is a subject of great expectations and gives rise to enormous hopes for patients who display severe forms of diseases without effective treatment. In the CVD field, MSCs-based therapy might be an advantageous alternative to current approaches and in the last decade, its potential has been demonstrated in numerous preclinical studies and it is being evaluated in clinics with promising results. Interestingly, the potential of MSCs to contribute to tissue repair has been found largely dependent on their secretory capacity (Figure 1) rather than their differentiation capacity. Thus far research has mostly focused on the secretion of cytokines and growth factors by MSCs. However, recent data suggests that the therapeutic effect of MSCs secretome can be partly due to secreted EVs, which can mirror the phenotype of their parent cells.

Therefore, the employment of cell-derived secretome to replace stem cells transplantation is of enormous interest. While MSCs are considered relatively safe, the development of therapeutic strategies that may avoid administration of living stem cells will attenuate the safety concerns relative to cell origin and immunocompatibility issues. A secretome-based approach should also minimise biological variability, allow precise dosing, and thus lead to the development of safe and effective therapeutic strategies with possibly predictable outcomes. Another advantage is the possibility of avoiding the lung barrier, one of the major obstacles for systemic administration of MSCs. Finally, given that patients with HF are at an increased surgical risk, the development of a noninvasive therapeutic approach looks very appealing.

In this perspective, the possibility of harnessing the MSCs secretome (both soluble factors and EVs) would have certain advantages over administration of a single factor that cannot mimic the actions of MSCs. Several questions have however to be addressed before clinical use can be considered.

## Figures and Tables

**Table 1:** Summary of molecules released by MSCs, via either direct secretion or exosomes and microvesicles, and their diverse beneficial effects in cardiovascular repair. The table includes relevant studies demonstrating specific molecule expression/secretion by MSCs and their respective effects.

Way of secretion	Molecule	Functional role in cardiovascular repair	References
Direct secretion	*Adrenomedullin *	Angiogenic and cardioprotective factor	[13]
	*Angiogenin *	LV remodeling attenuation through vasculogenesis	[31]
	*Basic fibroblast growth factor * (*bFGF, FGF-2*)	Vascular regeneration and attenuation of apoptotic pathways, leading to reduced remodeling	[32]
	*CXCL12 *	Endothelial tube formation	[33]
	*Cystatin C *	Fibroblast chemoattraction	[34]
	*Cysteine-rich angiogenic inducer* (*Cyr61*)	Angiogenesis promotion	[35]
	*Dickkopf-related proteins * (*Dkk*)	JNK signaling activation, eventually resulting in cardiomyogenesis	[36]
	*ECM structural proteins *	Fibroblast chemoattraction	[34]
	*Hepatocyte growth factor* (*HGF*)	Mobilization of cardiac progenitor cells	[13]
	*Hypoxic induced Akt regulated stem cell factor* (*HASF*)	Cardiomyocytes survival improvement	[37]
	*Insulin-like growth factor* (*IGF*)	Antiapoptotic effect, angiogenesis promotion, and activation of resident CSCs	[38]
	*Interleukin-1* (*IL-1*)	Angiogenesis promotion	[39]
	*Interleukin-6* (*IL-6*)	VEGF induction	[13, 39]
	*Leukemia inhibitory factor* (*LIF*)	Mobilization of BM-progenitor cells and cardioprotection promotion	[40]
	*Pigment epithelium-derived factor* (*PEDF*)	Fibroblast chemoattraction	[34]
	*Placental growth factor* (*PLGF*)	Prevention of cell death of cardiomyocytes and endothelial cells	[13]
	*Secreted Frizzled Related Protein* (*SFRP 2*)	Fibrosis and apoptosis reduction, promotion of MSC self-renewal and engraftment	[41, 42]
	*Soluble TNFR1* (*sTNFR1*)	Inflammatory response attenuation	[43]
	*Stem cell-derived factor* (*SDF-1*)	Stem cell recruitment and cardiomyocyte and MSC survival	[32, 44]
	*TNF-α stimulated gene-6* (*TSG-6*)	Anti-inflammation action	[17]
	*Vascular endothelial growth factor* (*VEGF*)	Prevention of cell death of cardiomyocytes and endothelial cells	[13]

Exosomes	*miR-19a *	Akt/ERK signaling activation, through PTEN targeting	[45]
	*miR22 *	Apoptosis reduction and ischemic CMCs injury improvement, through Mecp2 targeting	[46]
	*20S proteasome subunits* (*PMSA 1-7*)	Cardioprotection through proteolytic degradation of misfolded proteins	[47]
	*Unknown *	PMEC migration and vascularization improvement	[48]
	*Unknown *	Angiogenesis promotion	[49]
	*Unknown *	VEGF level incensement and angiogenesis promotion	[50]
	*Unknown *	Oxidative stress inhibition, PI3K/Akt pathway activation and inflammatory activity reduction	[51]
	*Unknown *	Hypoxic signal pathway inhibition	[52]

Microvesicles	*miR221 *	CMCs apoptosis reduction and CMCs survival improvement	[53]
	*Unknown *	Endothelial cell proliferation and blood flow recovery	[54]
